# Cross-Sectional Study of Patients With Onset of Acute Coronary Syndrome During Statin Therapy

**DOI:** 10.14740/jocmr2113w

**Published:** 2015-03-01

**Authors:** Nobuhiro Akuzawa, Takashi Hatori, Kunihiko Imai, Yonosuke Kitahara, Masahiko Kurabayashi

**Affiliations:** a Department of Internal Medicine, Gunma Chuo Hospital, 1-7-13 Koun-cho, Maebashi, Gunma 371-0025, Japan; b Department of Medicine and Biological Science, Gunma University Graduate School of Medicine, 3-39-22 Showa-machi, Maebashi, Gunma 371-8511, Japan

**Keywords:** Acute coronary syndrome, Low-density lipoprotein cholesterol, Statin, Triglyceride

## Abstract

**Background:**

Although statin therapy significantly reduces cardiovascular morbidity and mortality, atherosclerotic plaque progresses in some patients taking statins. This study investigated the factors associated with onset of acute coronary syndrome (ACS) early after the initiation of statin therapy.

**Methods:**

Consecutive patients taking statins who presented with ACS (n = 64) were divided into < 1-year and > 1-year groups based on the duration of statin therapy. Patient characteristics, coronary risk factors, lesion locations, and percutaneous intervention procedures were compared between groups.

**Results:**

The < 1-year group was significantly younger (57.6 ± 11.9 years vs. 76.6 ± 9.1 years, P < 0.01), had significantly higher body mass index (27.22 ± 4.20 kg/m^2^ vs. 24.60 ± 4.65 kg/m^2^, P < 0.05), higher proportion of males (94% vs. 70%, P < 0.05), higher proportion of current smokers (61% vs. 17%, P < 0.01), and lower proportions taking aspirin and calcium antagonists (both 17% vs. 57%, P < 0.05) than the > 1-year group. In the < 1-year group, there were significant correlations between the low-density lipoprotein cholesterol (LDL-C) and triglyceride (TG) levels (r = 0.649, P = 0.004) and between the TG and hemoglobin (Hb)A1c levels (r = 0.552, P = 0.018), but these correlations were not observed a year before admission. TG level was the only parameter associated with LDL-C and HbA1c levels.

**Conclusions:**

A linear correlation between the LDL-C and TG levels, obesity, older age, male sex, and smoking may be associated with increased risk of onset of ACS early after the initiation of statin therapy. Prospective cohort studies are needed to further explore these interactions.

## Introduction

The 3-hydroxy-3-methylglutaryl coenzyme A reductase inhibitors, known as statins, are effective for both primary and secondary prevention of cardiovascular disease (CVD) [1, 2]. Statins decrease the low-density lipoprotein cholesterol (LDL-C) level, and have anti-inflammatory and immunomodulatory effects on atherosclerotic plaque [3-5].

In patients undergoing intensive lipid-lowering therapy with atorvastatin, decreased coronary plaque volume showed a significant positive correlation with the percent decrease in LDL-C level [6]. Some studies that used virtual histology-intravascular ultrasound to follow patients found that coronary plaque regression after statin treatment correlated with a prominent decrease in the fibrofatty component within a few weeks and lasted for 8 - 9 months [7-10]. However, coronary plaques may progress despite early statin treatment and a subsequent low LDL-C level in patients with acute coronary syndrome (ACS) [7]. The specific lipid profiles associated with plaque progression despite statin therapy are currently unclear.

The primary objective of this study was to investigate the risk factors and lipid profiles in patients with onset of ACS < 1 year after the initiation of statin therapy.

## Materials and Methods

### Subjects

A total of 167 patients (119 males and 48 females) who underwent emergent percutaneous coronary intervention (PCI) after admission to our hospital with their first presentation of ACS between January 2009 and December 2013 were identified in our department database. Sixty-four of these patients (49 males and 15 females) were taking statins at admission, and were included in this study. The study was approved by the Gunma Chuo Hospital Ethics Committee. Written informed consent for inclusion in the study was obtained from all enrolled patients.

### Clinical and laboratory findings

Patients were divided into two groups according to the period of statin therapy before admission for ACS: a < 1-year group (n = 18) and a > 1-year group (n = 46). All patients complained of chest pain or discomfort at rest within 48 h before admission, and had been diagnosed with a high LDL-C level (≥ 140 mg/dL) before statin administration. The infarct type was classified as ST-elevation myocardial infarction if there were both ST segment elevation on electrocardiography and an increase in the serum troponin I level to ≥ 0.05 ng/mL, or non-ST elevation ACS in all other cases. Known coronary risk factors including diabetes mellitus, dyslipidemia, hypertension, hyperuricemia, and current smoking were determined by interview and by review of the medical notes. Patients were also screened for previously undiagnosed disease as follows. Hypertension was diagnosed if the systolic blood pressure was > 140 mm Hg or diastolic blood pressure was > 90 mm Hg, according to the Japanese Society of Hypertension guidelines for the management of hypertension (JSH2014) [11]. Dyslipidemia was diagnosed if the triglyceride (TG) level was ≥ 150 mg/dL, the high-density lipoprotein cholesterol (HDL-C) level was < 40 mg/dL, or the LDL-C level was ≥ 140 mg/dL, according to the Japan Atherosclerosis Society guidelines for the diagnosis and prevention of atherosclerotic cardiovascular diseases, 2012 version [12]. Hyperuricemia was diagnosed if the uric acid (UA) level was ≥ 7.0 mg/dL, according to the Japanese guideline for the management of hyperuricemia and gout [13]. Diabetes mellitus was diagnosed if the hemoglobin (Hb)A1c level was ≥ 6.5% in addition to a fasting plasma glucose level of ≥ 126 mg/dL, or a 75-g oral glucose tolerance test 2-h glucose level of ≥ 200 mg/dL, or a random glucose level of ≥ 200 mg/dL, according to the Japan Diabetes Society evidence-based practice guideline for the treatment of diabetes in Japan 2013 [14]. The HDL-C, LDL-C, and TG levels were determined from fasting whole blood and plasma samples taken soon after admission. The HbA1c, UA, and C-reactive protein (CRP) levels were measured from plasma samples taken at admission. Any history of cerebral infarction or atrial fibrillation resulting in anti-platelet or anti-coagulant therapy prior to admission was recorded. Current medications were confirmed by pharmacists who checked all medications that patients brought to the hospital. Statins were classified as strong statins (atorvastatin, pitavastatin, and rosuvastatin) or standard statins (fluvastatin, pravastatin, and simvastatin). Body mass index (BMI) and laboratory data from a year before admission, including the HDL-C, LDL-C, TG, HbA1c, UA, and CRP levels, were also recorded.

### Coronary angiography and evaluation of the stenotic lesion

Cardiac catheterization was performed via a femoral or brachial artery using a 6- or 7-Fr sheath and catheters. After injection of isosorbidedinitrate (2 mg) into the coronary artery, angiography was performed using the AXIOM Artis dTA system (Siemens, Munich, Germany). The angiograms were retrospectively reviewed for this study, and all quantitative angiographic measurements were performed by the same physician who was blinded to the patient details. The percent stenosis was calculated based on the diameter of the culprit lesion and the reference diameter of the adjacent normal vessel. Evaluation of the target lesion by intravascular ultrasonography (Visiwave or Intrafocus II, Terumo, Tokyo, Japan) was attempted prior to PCI in all patients. The intravascular diameter measured by intravascular ultrasonography was used to choose appropriate stent and balloon sizes. Successful coronary stenting was defined as a minimum stenosis diameter of < 10% without major in-hospital complications [15]. Successful balloon angioplasty was defined as a minimum stenosis diameter of < 50% with final TIMI flow grade 3 and no side branch loss, flow-limiting dissection, or angiographic thrombus [15].

### Statistical analysis

Continuous data are presented as mean ± SD or number (%). Comparisons of the admission data between the < 1-year and > 1-year groups were performed using the unpaired *t* test for parametric data or the Mann-Whitney U test for non-parametric data. Non-parametric data, such as the number of patients, were compared between the two groups using the χ^2^ test. Comparisons within the same group were performed using the paired *t* test for parametric data, or the Wilcoxon signed rank test for non-parametric data. Pearson’s correlation coefficient was used to analyze correlations between different lipid parameters including the HDL-C, LDL-C, and TG levels, or between lipid parameters and other variables associated with CVD such as the HbA1c, UA, and CRP levels. A value of P < 0.05 was considered to indicate a statistically significant association on univariate analysis. As there were significant correlations between the LDL-C and TG levels and between the HbA1c and TG levels in the < 1-year group, the relationship of each variable with the LDL-C and HbA1c levels in this group was determined using multivariate stepwise linear regression analysis. Multicollinearity was assessed using the variance inflation factor (VIF). A VIF exceeding 10 indicates serious multicollinearity, and a value greater than 4 may be a cause for concern. The variables that were found to be significantly associated on univariate analyses (age, BMI, and HDL-C, LDL-C, TG, HbA1c, UA, and CRP levels) were included in the multivariate stepwise linear regression analyses, with a value of P < 0.05 considered statistically significant. The data at 1 year before admission (including BMI and the HDL-C, LDL-C, TG, HbA1c, UA, and CRP levels) were compared between the two groups as described above. For these data, Pearson’s correlation coefficients were also calculated. All analyses were performed using SPSS version 21.0J for Windows (SPSS, Chicago, IL).

## Results

The characteristics of patients in the < 1-year and > 1-year groups are shown in Table 1. Patients in the < 1-year group were significantly younger (57.6 ± 11.9 years vs. 76.6 ± 9.1 years, P < 0.01), had a significantly higher BMI (27.22 ± 4.20 kg/m^2^ vs. 24.60 ± 4.65 kg/m^2^, P < 0.05), and were more likely to be male (94% vs. 70%, P < 0.05) than patients in the > 1-year group. There were no significant differences in infarct type, coronary risk factors, or associated conditions between the two groups, except that the proportion of current smokers was significantly higher in the < 1-year group (61% vs. 17%, P < 0.01). Patients in the < 1-year group were significantly less likely to be taking aspirin (17% vs. 57%, P < 0.01) or a calcium antagonist (17% vs. 57%, P < 0.01), and were significantly more likely to be taking a strong statin (89% vs. 48%, P < 0.01) or rosuvastatin (44% vs. 15%, P < 0.05) than patients in the > 1-year group. Review of medications (other than statins) initiated within 1 year before admission showed that angiotensin-receptor blockers and sulfonylureas were initiated in two patients in the < 1-year group, and calcium antagonists, angiotensin-receptor blockers, and sulfonylureas were initiated in five patients in the > 1-year group.

**Table 1:** Patient Characteristics

	< 1-year statin therapy (n = 18)	> 1-year statin therapy (n = 46)	P
Age (years)	57.6 ± 11.9	76.6 ± 9.1**	< 0.001**
Male/female	17/1 (94/6)*	32/14 (70/30)	0.030*
BMI	27.22 ± 4.20*	24.60 ± 4.65	0.016*
Disease type			
STEMI	9 (50)	30 (65)	0.262
NSTE-ACS	9 (50)	16 (35)	0.262
Risk factors			
Diabetes mellitus	8 (44)	22 (48)	0.807
Hypertension	9 (50)	34 (74)	0.067
Hyperuricemia	3 (17)	4 (9)	0.305
Hypertriglyceridemia	4 (22)	7 (15)	0.370
Current smoking	11 (61)**	8 (17)	0.001**
Associated diseases			
History of CI	2 (11)	20 (43)*	0.014*
History of AF	1 (6)	6 (13)	0.358
Medications			
Aspirin	3 (17)	26 (57)**	0.004**
Clopidogrel	0 (0)	3 (7)	0.364
Ticlopidine	0 (0)	3 (7)	0.364
Warfarin	1 (6)	3 (7)	0.687
ARB/ACEI	11 (61)	27 (59)	0.860
β-blocker	1 (6)	7 (15)	0.277
Calcium antagonist	3 (17)	26 (57)**	0.004**
Sulfonylurea	3 (17)	7 (15)	0.579
Insulin	3 (17)	8 (17)	0.630
Strong statins	16 (89)**	22 (48)	0.003**
Atorvastatin	3 (17)	10 (22)	0.470
Pitavastatin	5 (27)	5 (11)	0.101
Rosuvastatin	8 (44)*	7 (15)	0.018*
Standard statins	2 (12)	24 (52)**	0.003**
Fluvastatin	1 (6)	7 (15)	0.277
Pravastatin	1 (6)	12 (26)	0.061
Simvastatin	0 (0)	5 (11)	0.180
Duration of statin therapy (years)	0.61 ± 0.27**	8.35 ± 4.65	< 0.001**

Values are n (%) or mean ± SD. *P < 0.05, **P < 0.01. BMI: body mass index; STEMI: ST-elevation myocardial infarction; NSTE-ACS: non-ST elevation acute coronary syndrome; CI: cerebral infarction; AF: atrial fibrillation; ARB: angiotensin-receptor blocker; ACEI: angiotensin-converting enzyme inhibitor.

Laboratory data on admission including the HDL-C, LDL-C, TG, HbA1c, UA, and CRP levels, locations of culprit lesions, and PCI procedures are shown in Table 2. The UA level was significantly higher in the < 1-year group than in the > 1-year group (5.81 ± 0.85 mg/dL vs. 5.05 ± 1.17 mg/dL, P < 0.05). PCI was unsuccessful in three patients in the > 1-year group, because the guidewire or the balloon could not be advanced through the stenosis. The locations of culprit lesions and the PCI procedures were not significantly different between the two groups. The only significant change in laboratory data between a year before admission and admission in either group was a significant decrease in the LDL-C level in the < 1-year group (P < 0.01).

**Table 2:** Comparisons of Laboratory Data, Culprit Lesions, and PCI Procedures at Admission

	< 1-year statin therapy (n = 18)	> 1-year statin therapy (n = 46)	P
Laboratory data			
HDL-C (mg/dL)	48.9 ± 8.8	49.1 ± 13.4	0.565
LDL-C (mg/dL)	124.8 ± 32.0	111.6 ± 29.5	0.122
TG (mg/dL)	186.1 ± 114.0	133.7 ± 79.4	0.064
HbA1c (%)	6.82 ± 1.78	6.65 ± 1.26	0.590
Uric acid (mg/dL)	5.81 ± 0.85*	5.05 ± 1.17	0.016*
CRP (mg/dL)	0.17 ± 0.14	0.21 ± 0.19	0.421
Culprit lesion			
RCA	4 (22)	14 (30)	0.511
LAD	12 (67)	20 (44)	0.095
LCX	2 (11)	12 (26)	0.168
% Stenosis	88.4 ± 9.1	88.2 ± 7.4	0.828
PCI procedure			
POBA alone	1 (6)	3 (7)	0.687
Stenting	17 (94)	40 (86)	0.358
Bare-metal stent	4 (22)	11 (24)	0.583
Drug-eluting stent	13 (72)	29 (63)	0.487
Mean stent diameter (mm)	3.23 ± 0.50	3.29 ± 0.44	0.693
Mean stent length (mm)	21.6 ± 5.8	20.7 ± 4.0	0.781
Unsuccessful PCI	0 (0)	3 (7)	0.364

Values are n (%) or mean ± SD. *P < 0.05. HDL-C: high-density lipoprotein cholesterol; LDL-C: low-density lipoprotein cholesterol; TG: triglyceride; HbA1c: hemoglobin A1c; CRP: C-reactive protein; RCA: right coronary artery; LAD: left anterior descending branch; LCX: left circumflex branch; POBA: plain old balloon angioplasty.

### Correlations between variables

**Figure 1:**
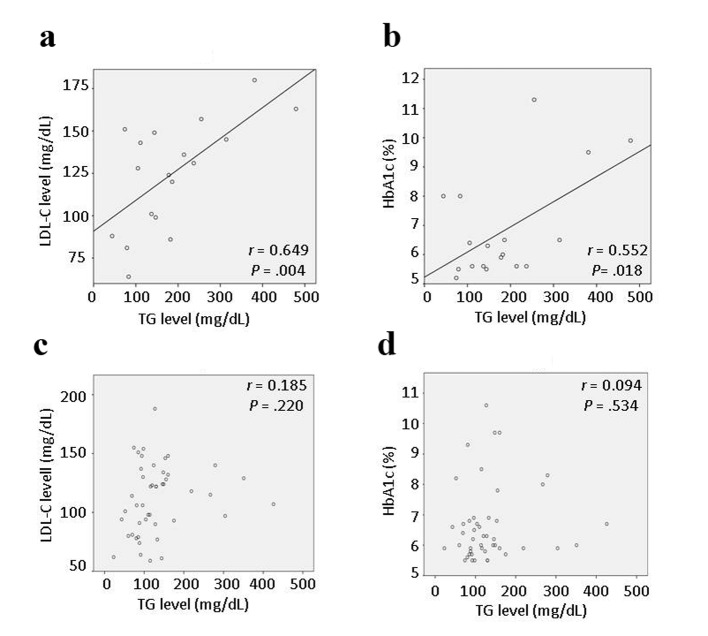
Correlations between the low-density lipoprotein cholesterol (LDL-C) and triglyceride (TG) levels, and between the hemoglobin (Hb)A1c and TG levels, at admission. In the < 1-year group, the TG level was significantly correlated with the LDL-C level (r = 0.649, P = 0.004) (a) and the HbA1c level (r = 0.552, P = 0.018) (b). In the > 1-year group, there was no significant correlation between the TG and LDL-C levels (c), or between the TG and HbA1c levels (d).

Although the initial comparisons did not show significant differences in laboratory data between the < 1-year and > 1-year groups, the specific correlations that could be affected by statin therapy were investigated further. Interestingly, in the < 1-year group, there were significant correlations between the LDL-C and TG levels (r = 0.649, P = 0.004), and between the HbA1c and TG levels (r = 0.552, P = 0.018) (Fig. 1a, b), but there were no significant correlations among other background characteristics including age, BMI, and HDL-C, UA, and CRP levels. Multivariate stepwise linear regression analyses also showed significant associations between the LDL-C and TG levels, and between the HbA1c and TG levels (Table 3). There was no evidence of multicollinearity (the VIF for independent variables was < 2.0). The multiple regression coefficient (R) and coefficient of determination (R^2^) were 0.649 and 0.421 for the association between the LDL-C and TG levels, and 0.552 and 0.304 for the association between the HbA1c and TG levels, respectively. In the > 1-year group, there were no significant correlations between different lipid parameters including the HDL-C, LDL-C and TG levels, or between lipid parameters and other variables associated with CVD (Fig. 1c, d).

**Table 3:** Multivariate Stepwise Linear Regression Analyses for Factors Associated With the LDL-C and HbA1c Levels in Patients With Onset of ACS <1 Year After the Initiation of Statin Therapy

Factors associated with LDL-C level				
Independent variable	B (95% CI)	β	*t*	P
TG	0.18 (0.07 - 0.30)	0.649	3.413	0.004
Factors associated with HbA1c level				
Independent variable	B (95% CI)	β	*t*	P
TG	0.009 (0.002 - 0.015)	0.552	2.646	0.018

B: non-standardized regression coefficient; CI: confidence interval; β: standardized regression coefficient.

BMI and laboratory data a year before admission, including the HDL-C, LDL-C, TG, HbA1c, UA and CRP levels, were analyzed in the same manner as admission data (< 1-year group: n = 17, > 1-year group: n = 37) (Table 4). The BMI and LDL-C level a year before admission were significantly higher in the < 1-year group than in the > 1-year group, reflecting the absence of statin therapy in the < 1-year group. The UA level a year before admission was also significantly higher in the < 1-year group than in the > 1-year group, showing the same pattern as on admission. Comparisons of data between a year before admission and admission within each group showed that the LDL-C level was significantly higher a year before admission than at admission in the < 1-year group (P < 0.01). There were no significant correlations between different lipid parameters including the HDL-C, LDL-C and TG levels, or between lipid parameters and other variables associated with CVD, in either group. In particular, the TG level was not correlated with the LDL-C or HbA1c level in either group.

**Table 4:** Laboratory Data a Year Before Admission

	< 1-year statin therapy (n = 17)	> 1-year statin therapy (n = 39)	P
BMI	27.58 ± 4.40*	24.65 ± 4.78	0.035*
HDL-C (mg/dL)	52.0 ± 10.7	51.0 ± 14.0	0.605
LDL-C (mg/dL)	166.5 ± 25.1**	115.9 ± 29.5	< 0.001**
TG (mg/dL)	186.3 ± 68.0	150.2 ± 98.3	0.175
HbA1c (%)	6.98 ± 2.15	6.50 ± 1.19	0.872
Uric acid (mg/dL)	5.75 ± 1.13*	4.95 ± 1.09	0.015*
CRP (mg/dL)	0.11 ± 0.08	0.22 ± 0.40	0.652

Values are n (%) or mean ± SD. *P < 0.05, **P < 0.01. HDL-C: high-density lipoprotein cholesterol; LDL-C: low-density lipoprotein cholesterol; TG: triglyceride; HbA1c: hemoglobin A1c; CRP: C-reactive protein.

## Discussion

This study focused on patients with onset of ACS despite statin therapy to treat dyslipidemia and lower the CVD risk. Among the 64 patients taking statins who were admitted with ACS, 18 had started statin therapy within the past year. The < 1-year group had a significantly younger age, higher proportion of males, higher BMI, and higher proportion of current smokers than the > 1-year group. Obesity and smoking are well-known risk factors for CVD. Obesity deteriorates vascular inflammation in relation to hypoadiponectinemia [16]. The extent of coronary artery lesions is comparable between smokers and non-smokers with coronary artery disease, but the lesions develop 10 years earlier in smokers [17]. The younger age of the < 1-year group may reflect the contributions of these risk factors. Our data suggest that lipid-lowering therapy using statins alone may be insufficient to prevent the early onset of ACS, especially in obese males with high LDL-C levels. Moreover, the significantly lower proportions of patients taking aspirin and calcium antagonists, or higher UA level in the < 1 year group may affect the early ACS onset after statin therapy. However, patients’ characteristics of the < 1-year group, such as higher proportion of smokers or males, and higher BMI, correspond to common risk factors of CAD and thus it is difficult to say that these characteristics may be crucial for the early onset of ACS after statin therapy.

Past studies have reported a positive association between the TG level and CAD [18, 19]. In the present study, there was a significant linear correlation between the TG and LDL-C levels on admission in the < 1-year group. Similar correlations have not been reported in patients with ACS or patients taking statins. The lowering effect of statins on the TG content of very-low-density lipoprotein (VLDL) is weaker than the lowering effect on LDL-C [20]. In rat and mouse models, treatment with statins decreased the serum TG level and hepatic VLDL secretion, and increased biliary lipid secretion [21-23]. In human, atorvastatin can decrease the production or facilitate the catabolism of apolipoprotein (apo) B-100 in VLDL, intermediate-density lipoprotein (IDL), and LDL, and of apo B-48 in TG-rich lipoproteins [24]. It is notable that the mean TG levels were similar (≥ 150 mg/dL) at admission and a year before admission in the < 1-year group, suggesting that statin therapy may not have had much effect on the TG level in this group. This contrasts with the significant decrease in the LDL-C level during this period. Interestingly, there was no significant correlation between the TG and LDL-C levels in either group a year before admission, before the introduction of statin therapy in the < 1-year group. Considering that medication changes occurred in only a few patients in each group, these results indicate that the initiation of statin therapy may affect the correlation between the TG and LDL-C levels. As the LDL-C level is measured by the β-quantification method at our institution, IDL-cholesterol is included in the LDL-C level [25]. The linear correlation between the TG and LDL-C levels in the < 1-year group may therefore reflect changes in the lipid compositions of lipoproteins, resulting in formation of TG-rich or TG-saturated LDL and IDL with decreased cholesterol content. In addition, the higher proportion of patients taking strong statins in the < 1-year group may have resulted in a greater decrease in cholesterol content and greater increase in TG content in LDL and IDL. This remains speculative, because the TG and cholesterol levels in LDL and IDL were not measured in this study. But the results of a previous study showing that high-dose administration of atorvastatin did not induce a significant reduction in TG content of LDL [26], support our hypothesis and thus an increased TG content of LDL or IDL may be associated with onset of ACS early after the initiation of statin therapy. Previously reported data suggest that higher TG level may increase the risk of CVD partly because of the accompanying burden of atherogenic remnant particles, small dense LDL, reduced HDL-C, and a high frequency of insulin resistance [27]. The present study also found a stronger correlation between the HbA1c and TG levels compared to a previous study [28]. This finding suggests that statin therapy may increase the influence of TG on glycometabolism, but the underlying mechanisms are currently unknown.

Taken together, detection of a correlation between the LDL-C and TG levels may be a novel predictive marker for onset of ACS early after the initiation of statin therapy. Our findings also suggest that older male smokers with persistent hypertriglyceridemia and low LDL-C levels after the initiation of statin therapy may have a high risk of ACS onset. However, effective treatment for reducing the CVD risk by regulating both the LDL-C and TG level has not been established. Further investigation of add-on medications is needed to enable more effective primary prevention of CVD.

### Limitations

This study is limited by the relatively small number of patients from a single institution, possible selection bias, and the retrospective cross-sectional design. The relationship between onset of ACS and strong statin use is therefore unclear. Similarly, the preventive effects of other medications such as aspirin and calcium antagonists on onset of ACS after the initiation of statin therapy could not be analyzed. In addition, the TG, cholesterol, apo B-100, and apo B-48 levels in LDL and IDL were not measured. Although there were significant correlations between the LDL-C and TG levels, and between the HbA1c and TG levels, the effects of these parameters on the onset of ACS and on plaque size and vulnerability are still unclear. It is also unclear whether the correlations between the LDL-C and TG levels, and between the HbA1c and TG levels, in the < 1-year group were also present in the group overall or in specific patients a year after the initiation of statin therapy, because data from before the initiation of statin therapy were not available for the > 1-year group. Prospective cohort studies are needed to further explore these interactions.

### Conclusions

Patients with onset of ACS < 1 year after the initiation of statin therapy had a significant linear correlation between the TG and LDL-C levels at admission, which was not observed a year before admission. Patients with onset of ACS < 1 year after the initiation of statin therapy had a higher BMI and were more likely to be male, current smokers, and strong statin users than patients with onset of ACS > 1 year after the initiation of statin therapy. These patients also had persistent hypertriglyceridemia after the initiation of statin therapy despite a significant decrease in the LDL-C level. Further investigation is needed to determine the underlying risk factors in patients with onset of ACS early after the initiation of statin therapy.
